# Thermal Modality-Based Treatment of Gingival Hyperpigmentation: A Case Report

**DOI:** 10.7759/cureus.83499

**Published:** 2025-05-05

**Authors:** Mehvish Khan, Shubham Sharma, Riya Agarwal, Mayur Kaushik, Roopse Singh

**Affiliations:** 1 Department of Periodontology and Implantology, Subharti Dental College and Hospital, Meerut, IND

**Keywords:** cosmetic dentsitry, diode laser therapy, esthetics dentistry, gingival hyperpigmentation, monopolar electrosurgery

## Abstract

Gingival hyperpigmentation, caused by excessive melanin deposition in the basal and suprabasal layers of the gingival epithelium, is a significant aesthetic concern, particularly for individuals with a high smile line. Although benign, this condition can negatively impact self-confidence, prompting many patients to seek cosmetic treatment. Various depigmentation techniques exist, including scalpel surgery, cryotherapy, electrosurgery, and laser therapy, each offering distinct advantages and limitations. This case report presents a comparative evaluation of diode laser and electrosurgery for gingival depigmentation using a split-mouth approach. The mandibular arch was treated with an 810 nm diode laser (Surgical Laser Clean Cut, 810NM/10W; Confident Dental Equipments, Bangalore, India), while the maxillary arch was treated using a monopolar electrosurgical unit (Younique Dental Innovations R.F. Advance; Motranser, Waipu, Taiwan). Clinical parameters assessed included healing time, intraoperative bleeding control, patient comfort, and recurrence rates over a six-month follow-up period. Findings suggest that while electrosurgery provided superior hemostasis, the diode laser led to faster healing and a lower recurrence rate. This study highlights the importance of selecting an appropriate depigmentation technique based on patient-specific needs, emphasizing the role of minimally invasive procedures in modern periodontal esthetics.

## Introduction

Gingival pigmentation is a common condition that results from melanin deposition in the basal and suprabasal layers of the epithelium. While physiologic pigmentation does not pose any pathological concerns, it can be an aesthetic issue, especially for patients with a high smile line [1]. Factors contributing to gingival pigmentation include genetic predisposition, racial background, smoking, systemic diseases, and chronic irritation from dental prostheses or restorations [2].

Several treatment modalities have been used for gingival depigmentation. Scalpel surgery is one of the oldest and most commonly performed techniques but is associated with significant postoperative discomfort, prolonged healing, and a higher likelihood of recurrence [3]. Chemical cauterization with phenol or alcohol has also been used but may lead to mucosal burns and uneven healing [4]. Yadav et al. [5] suggested cryotherapy, involving the use of liquid nitrogen or carbon dioxide, can remove pigmentation effectively, but the healing pattern is often unpredictable.

Electrosurgery and laser therapy have emerged as preferred methods due to their minimally invasive nature and enhanced precision. Electrosurgery utilizes high-frequency electrical currents to excise the pigmented epithelium while simultaneously coagulating blood vessels, ensuring excellent intraoperative hemostasis [6]. In contrast, El Shenawy et al. [7] stated that diode lasers selectively target melanocytes, allowing for precise ablation with minimal thermal damage to surrounding tissues, resulting in reduced postoperative discomfort and faster healing.

Diode laser and electrosurgery are both effective thermal techniques for gingival depigmentation but differ in tissue interaction and clinical outcomes. Comparing them allows evaluation of healing, patient comfort, bleeding control, and recurrence within the same patient, helping clinicians choose the most appropriate modality based on esthetic goals and clinical needs.

This case report aims to compare the effectiveness of diode laser and electrosurgical techniques in gingival depigmentation using a split-mouth approach. The study evaluates patient comfort, healing time, recurrence, and intraoperative bleeding control.

## Case presentation

**Figure 1:**
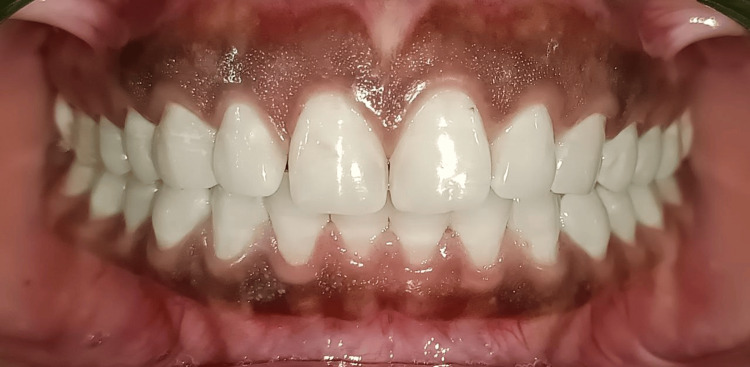
Pre-operative view

A 25-year-old male patient presented with generalized gingival hyperpigmentation and dissatisfaction with the darkened appearance of his gums (Figure 1).

Clinical examination classified the pigmentation as moderate to severe according to the Dummett-Gupta Oral Pigmentation Index (DOPI ≥2) [1]. The DOPI is a standardized clinical index used to assess the intensity of gingival pigmentation based on visual inspection. It scores pigmentation as follows: Score 0: No clinical pigmentation (pink gingiva); Score 1: Mild pigmentation (light brown tissue); Score 2: Moderate pigmentation (medium brown or a mix of pink and brown); Score 3: Heavy pigmentation (deep brown or bluish-black tissue).

This index provides a standardized method to evaluate baseline pigmentation and assess treatment outcomes in depigmentation procedures.

The patient had no history of systemic diseases, smoking, or medication use that could contribute to pigmentation. Given his esthetic concerns, a split-mouth approach was chosen to compare the efficacy of diode laser and electrosurgery for depigmentation.

**Figure 2:**
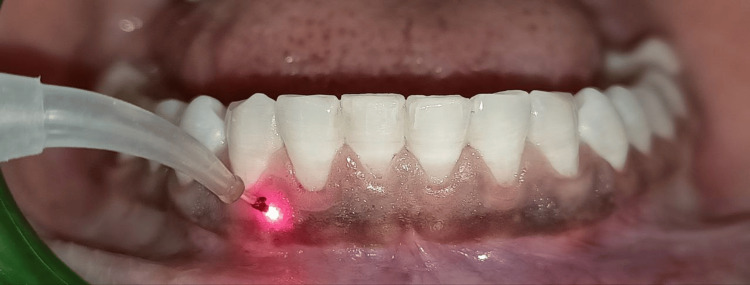
Diode laser application on mandibular arch

Local anesthesia (2% lignocaine with epinephrine) was administered before the procedure. The mandibular arch was treated with an 810 nm diode laser (SURGICAL LASER CLEAN CUT, 810NM/10W; Confident Dental Equipments, Bangalore, India) in continuous mode at 1.5 W using a 400-micron fiber tip, applied in a sweeping motion over the pigmented epithelium (Figure 2). Care was taken to prevent excessive heat buildup, minimizing collateral tissue damage.

**Figure 3:**
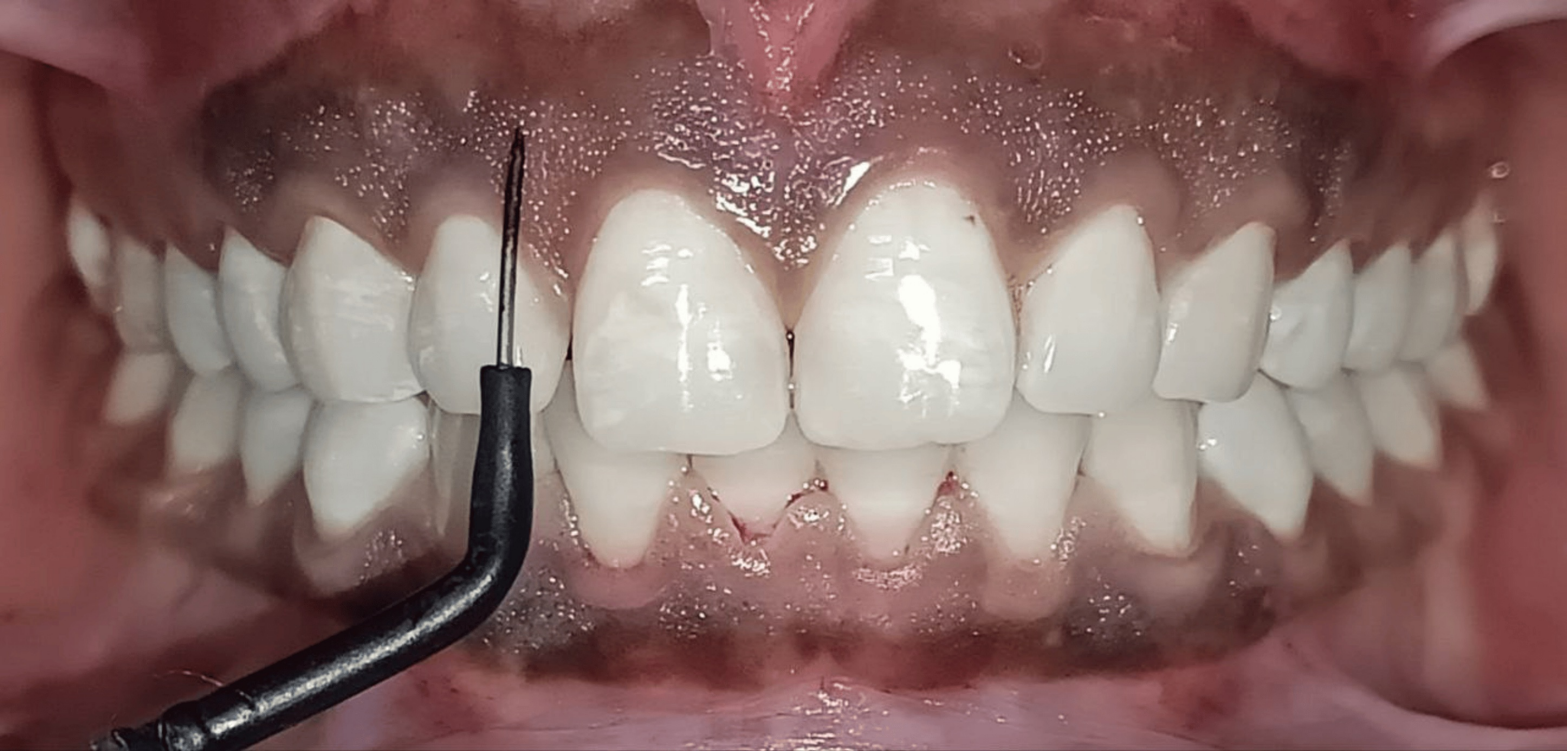
Electrosurgical unit application on maxillary arch

The maxillary arch was treated using a monopolar electrosurgical unit with a fine straight electrode (Younique Dental Innovations R.F. Advance; Motranser, Waipu, Taiwan), which allowed for controlled tissue removal while providing simultaneous coagulation to reduce intraoperative bleeding (Figure 3).

**Figure 4:**
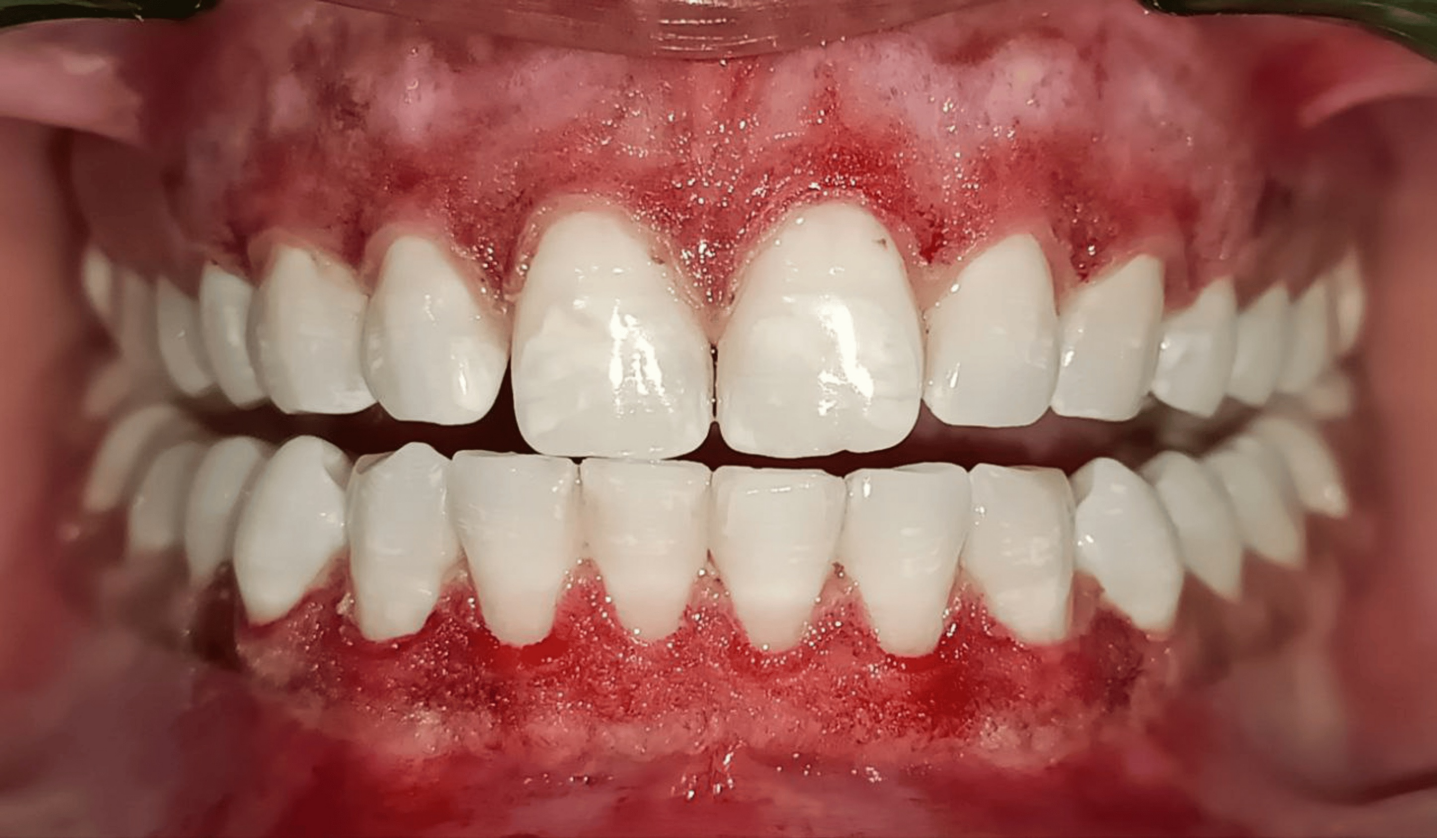
Immediate postoperative view

**Figure 5:**
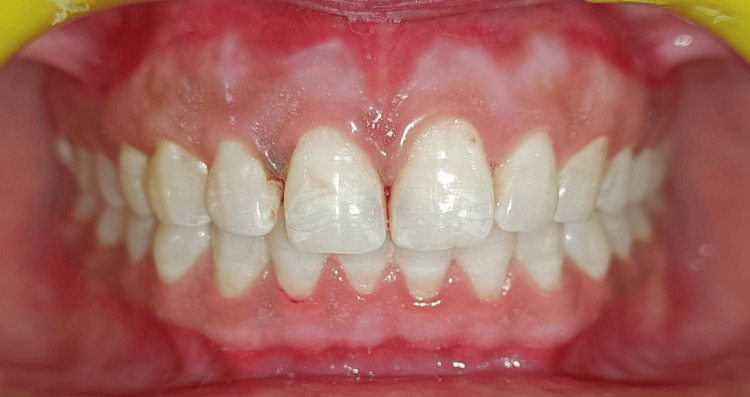
One-month postoperative view

**Figure 6:**
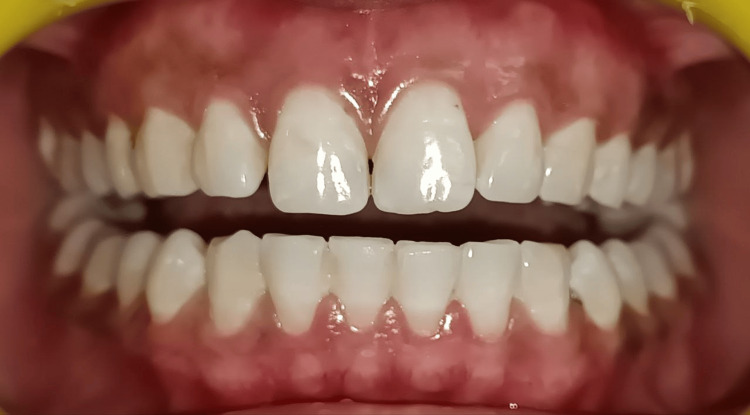
Three-month postoperative view

Postoperatively, the patient was instructed to maintain oral hygiene using a soft-bristle toothbrush and to avoid spicy or hot foods. Chlorhexidine (0.12%) mouthwash was prescribed to aid healing and prevent infections. Follow-up evaluations were conducted immediately after the procedure was completed (Figure 4), one month (Figure 5) and three months (Figure 6) to monitor healing and recurrence.

Healing outcomes varied between the two techniques. The diode laser-treated site healed within seven to 10 days with minimal inflammation, whereas the electrosurgical site required 10-14 days for complete epithelialization. Intraoperative bleeding was more effectively controlled with electrosurgery due to its simultaneous excision and coagulation properties. However, at the six-month follow-up, the diode laser-treated site remained depigmented with no recurrence, whereas mild pigmentation recurrence was observed in the electrosurgical-treated site.

## Discussion

Healing time and patient comfort are critical factors in evaluating depigmentation techniques. The diode laser-treated site healed within seven to 10 days, while the electrosurgery-treated site required 10 to 14 days. Healing time and patient comfort are critical factors in evaluating depigmentation techniques. The diode laser-treated site healed within seven to 10 days, while the electrosurgery-treated site required 10 to 14 days. This difference can be attributed to the mechanism of action of each modality. The diode laser delivers light energy at 810-980 nm, which is selectively absorbed by melanin-rich tissues. This results in precise photothermal ablation of pigmented epithelial cells with minimal collateral damage. Additionally, laser biostimulation enhances fibroblast activity and vascular regeneration, contributing to faster epithelialization and reduced postoperative inflammation. Cobb [8] reported that laser therapy enhances tissue regeneration by stimulating fibroblast activity and promoting faster epithelialization.

In contrast, electrosurgery employs high-frequency electrical currents to cut and coagulate tissue through thermal energy. The generated heat causes dehydration and protein denaturation in the targeted tissue, effectively removing pigmented epithelium. However, the lateral spread of this heat may inadvertently affect adjacent non-pigmented areas, leading to increased inflammation and a slower healing response. These biological differences align with the findings in our case, where diode laser resulted in quicker healing and better patient tolerance compared to electrosurgery. Romanos [9] noted that high-frequency electrical currents can extend beyond the target area, causing increased postoperative discomfort. This correlates with the present findings, where the electrosurgical-treated site exhibited slightly delayed healing and greater discomfort compared to the diode laser-treated site.

Bleeding control is another crucial aspect of depigmentation techniques. Electrosurgery provided superior intraoperative hemostasis due to its ability to cut and coagulate simultaneously, ensuring a nearly bloodless field and facilitating precise excision of pigmented epithelium. Tal et al. [10] demonstrated that electrosurgical depigmentation significantly reduces intraoperative bleeding, making it a valuable option for cases where excessive bleeding is a concern. 

Although diode lasers also offer coagulative benefits, their hemostatic effects depend on wavelength and power settings. Unlike electrosurgery, which instantly seals blood vessels through thermal coagulation, diode lasers require slightly longer exposure times to achieve similar results. This finding aligns with a study by Bakhshi et al. [11], which suggests that while lasers provide adequate hemostasis, they may not match the immediate coagulation efficiency of electrosurgery.

Laser therapy has proven to be effective in precisely targeting and removing epithelial cells, especially those at the base layer, which helps prevent the return of dark pigmentation better than other methods [12]. Arun et al [13], highlights the advantages of electrosurgery in managing bleeding and maintaining patient comfort requires precise tissue handling. The differential clinical outcomes observed between diode laser and electrosurgical modalities underscore the importance of understanding their distinct biological effects. The laser costs approximately three to five times more upfront than electrosurgery. Strategic selection of the depigmentation technique, grounded in evidence-based parameters such as tissue response, healing kinetics, and hemostatic efficacy, is essential to optimize therapeutic success and long-term esthetic stability. A table summarizing the basic clinical comparison between diode laser and electrosurgery is provided below (Table 1).

**Table 1:** Clinical Comparison of Diode Laser and Electrosurgery for Gingival Depigmentation

Parameter	Diode Laser (Mandibular Arch)	Electrosurgery (Maxillary Arch)
Power Settings	1.5 W, continuous mode, 400 μm tip	40% power, cut/coagulate mode
Healing Time	7–10 days	10–14 days
Postoperative Discomfort	Minimal	Moderate
Intraoperative Bleeding	Mild, controlled	Excellent hemostasis
Recurrence at 3 Months	No recurrence observed	Mild pigmentation recurrence
Patient Tolerance	High (well accepted)	Moderate (slightly more discomfort)

## Conclusions

This study demonstrates that both diode laser and electrosurgery are effective for gingival depigmentation. Diode lasers offer superior healing, reduced postoperative discomfort, and lower recurrence rates, making them ideal for long-term esthetic stability. Electrosurgery ensures excellent intraoperative bleeding control and remains a cost-effective alternative for cases where hemostasis is a priority. Treatment selection should be guided by patient needs, clinical objectives, and long-term esthetic considerations. This study is limited by its single-case design and the lack of standardized scoring measures, which may affect the generalizability of results. Larger, controlled studies are needed to validate these findings and support broader clinical application. In the pursuit of the perfect smile, precision, comfort, and clinical insight make all the difference.
